# Increased FUS levels in astrocytes leads to astrocyte and microglia activation and neuronal death

**DOI:** 10.1038/s41598-019-41040-4

**Published:** 2019-03-14

**Authors:** Maria Antonietta Ajmone-Cat, Angela Onori, Camilla Toselli, Eleonora Stronati, Mariangela Morlando, Irene Bozzoni, Emanuela Monni, Zaal Kokaia, Giuseppe Lupo, Luisa Minghetti, Stefano Biagioni, Emanuele Cacci

**Affiliations:** 1 National Center for Drug Research and Evaluation, Istituto Superiore di Sanità, Rome, Italy; 2 Department of Biology and Biotechnology “Charles Darwin”, Sapienza University of Rome, Rome, Italy; 3 Laboratory of Stem Cells & Restorative Neurology, Stem Cell Center, Lund University Hospital, Lund, Sweden; 4 Department of Chemistry, Sapienza University of Rome, Rome, Italy; 5 Research Coordination and Support Service, Istituto Superiore di Sanità, Rome, Italy; 6 Center for Life Nano Science, Istituto Italiano di Tecnologia, Rome, Italy

## Abstract

Mutations of Fused in sarcoma (FUS), a ribonucleoprotein involved in RNA metabolism, have been found associated with both familial and sporadic cases of amyotrophic lateral sclerosis (ALS). Notably, besides mutations in the coding sequence, also mutations into the 3′ untranslated region, leading to increased levels of the wild-type protein, have been associated with neuronal death and ALS pathology, in ALS models and patients. The mechanistic link between altered FUS levels and ALS-related neurodegeneration is far to be elucidated, as well as the consequences of elevated FUS levels in the modulation of the inflammatory response sustained by glial cells, a well-recognized player in ALS progression. Here, we studied the effect of wild-type FUS overexpression on the responsiveness of mouse and human neural progenitor-derived astrocytes to a pro-inflammatory stimulus (IL1β) used to mimic an inflammatory environment. We found that astrocytes with increased FUS levels were more sensitive to IL1β, as shown by their enhanced expression of inflammatory genes, compared with control astrocytes. Moreover, astrocytes overexpressing FUS promoted neuronal cell death and pro-inflammatory microglia activation. We conclude that overexpression of wild-type FUS intrinsically affects astrocyte reactivity and drives their properties toward pro-inflammatory and neurotoxic functions, suggesting that a non-cell autonomous mechanism can support neurodegeneration in FUS-mutated animals and patients.

## Introduction

Fused in sarcoma (FUS) or translocated in liposarcoma (TLS) is an ubiquitously expressed protein belonging to the family of heterogeneous nuclear ribonucleoproteins, continuously shuttling between the nuclear and cytoplasmic compartments, involved in pre-mRNA splicing, mRNA stability, and mRNA transport^1–3^.

*FUS* mutations have been identified in 4% of familial and 1% of sporadic amyotrophic lateral sclerosis (ALS) cases^4–6^. Moreover, *FUS* mutations are also associated with the ALS-related disorder frontotemporal dementia^7^.

Several mutations (e.g. *FUS* P525L, *FUS* P525R) affecting the C-terminus, lead to disruption of the nuclear localization signal, cause accumulation of FUS in the cytoplasm^8^, and are associated with a very aggressive and precocious form of ALS^9^. Of importance, mutations in the 3′ untranslated region (3′ UTR) of *FUS*, causing elevation of FUS in the wild-type (WT) configuration, have also been identified in a subset of ALS patients^10,11^.

Studies in different animal models indicate that increased expression of WT-FUS induces neurodegeneration^12^. In accordance with findings in yeast and drosophila^12–15^, overexpression of human WT-FUS in mice causes an aggressive ALS phenotype, with progressive neuron degeneration and astrogliosis^16^, suggesting that the excess of WT-FUS is toxic for motorneurons (MNs). Mechanisms by which overexpression of WT-FUS causes motor neuron degeneration remain to be elucidated.

Several lines of evidence suggest that MN degeneration results from the combination of cell intrinsic MN vulnerability^17,18^, and the toxic effects of inflammatory response during disease progression^19,20^. Astrocytes and microglia influence the progression of neurodegeneration in both ALS patients and mouse models of the disease^21–29^.

Gliosis with increased production of potentially harmful inflammatory mediators, including interleukin 1β (IL1β), tumour necrosis factor α (TNFα), interleukin 6 (IL6), prostanoids, and reactive oxygen species (ROS), was reported in the spinal cords of ALS patients^19^. In addition, upregulation of the transcription factor NF-kB, a master regulator of inflammation, was found in glial cells of both familial and sporadic ALS patients^30–32^.

FUS is involved in the pathogen responses in dendritic cells^33^ and it was shown to interact with NF-kB, specifically the p65 subunit^34^, suggesting that mutation disrupting *FUS* sequence or levels may affect this pathway and the immune function of specialized cells.

The link between neuroinflammation and MN degeneration has been extensively explored in different ALS subtypes, but represents a novel, almost unexplored issue, in relation to FUS.

Here, we analyzed the effects of elevated levels of WT-FUS on astrocyte functional properties, focusing on their response to a pro-inflammatory stimulus, and on their cross-talk with microglia and neuronal cells. We used mouse and human neural progenitor cells isolated from fetal spinal cord (mNPsc or hNPsc, respectively), to generate astrocytes expressing increased levels of WT-FUS, under the control of a doxycycline-inducible promoter. We found that several genes, including *IL6*, *TNF*α, prostaglandin-endoperoxide synthase 2 (*PTGS2)*, and related proteins or metabolites were more abundantly expressed in astrocytes overexpressing FUS, and stimulated with IL1β, indicating increased reactivity to this pro-inflammatory cytokine in comparison with control astrocytes. Furthermore, conditioned medium from cultured astrocyte-like cells overexpressing FUS potentiated pro-inflammatory microglia activation and increased neuronal cell death. Our data indicate that FUS upregulation modulates the expression of several inflammatory genes, possibly through NF-kB activation, and confers neurotoxic potential to astrocytes *in vitro*.

## Results

### Establishment of FUS-overexpressing astrocyte-like cells of spinal cord origin, and assessment of exogenous FUS localization within cell compartments

To investigate the functional consequences of WT-FUS overexpression in spinal cord astrocytes, we took advantage of the well-characterized model of mNP_SC_ that can be efficiently differentiated into astrocyte-like cells, when treated with the morphogen BMP-4, as previously described by us and others^35–40^. The astrocytic differentiation under these conditions is further corroborated by the up-regulation of typical astrocyte differentiation markers, such as GFAP, S100b, Aquaporin-4, FGF3 and Etv5, upon BMP-4 treatment, as reported in Supplemental Fig. 1.

mNPsc were engineered with plasmids allowing for the expression of human WT-FUS under doxycycline (Dox) control^41^, and differentiated into astrocyte-like cells by adding 25 ng/ml BMP-4 for 6 days, in the presence or in the absence of the selected dose of 50 ng/ml Dox (see Materials and Methods for details).

**Figure 1:**
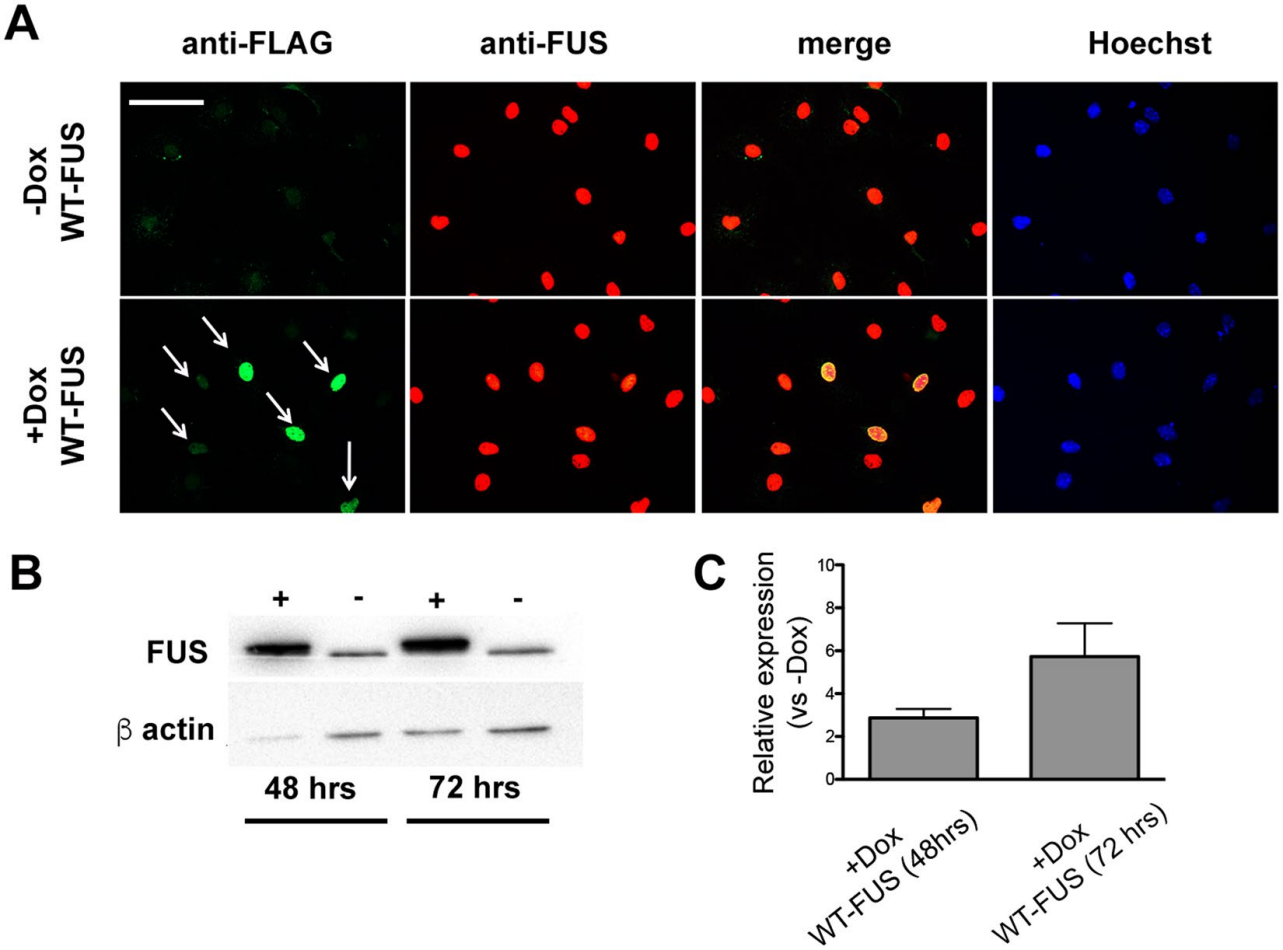
Expression of FUS in NP-derived astrocytes. mNPsc electroporated with plasmids allowing for the conditional expression of human WT-FUS were maintained for six days in the presence of astrocyte differentiation medium supplemented with or without doxycycline (Dox) and then fixed and stained. (**A**) Exogenous FUS, identified by an anti-FLAG antibody, was localized in the nucleus (WT-FUS) only in cells exposed to Dox. Similar staining profile was obtained by using an antibody recognizing both endogenous and the exogenous FUS (anti-FUS antibody). Scale bar 30 μm. (**B**,**C**) Representative blot and quantification of relative FUS levels at 48 and 72 hrs upon doxycycline stimulation. The bar graph shows a significant increase of FUS expression in induced cells (+Dox) compared to control cells (−Dox). The band observed in the lanes −Dox reflects endogenous FUS expression. Values are expressed as the mean of two independent experiments. FUS levels are normalized with respect to β-actin.

The transgenic protein, identified by immunostaining with an anti-Flag antibody, was mainly localized within nuclei, and its expression was tightly dependent on Dox treatment (Fig. 1A). The overexpression of the WT form did not significantly alter its subcellular localization.

As shown by Western blot, WT-FUS cells, administered with Dox for 48 and 72 hrs, showed a time-dependent increase of FUS levels of about 2.5- and 6-folds, respectively, compared to non-induced cells (Fig. 1B,C; see also Supplemental Fig. 2 for the original blot).

### Elevation of WT-FUS modulates the expression of several inflammatory genes and increases NF-kB activation in response to IL1β

In order to analyze whether FUS overexpression determined astrogliosis, even in the absence of an inflammatory stimulus, we analyzed the accumulation in the culture media of relevant inflammatory proteins/metabolites usually upregulated in the inflammatory activation state. We focused on the free radical nitric oxide (NO), produced by the inducible enzyme iNOS, PGE_2_ - a major prostanoid produced by the inducible enzyme PTGS2 during brain inflammation^42^ -, and the pro-inflammatory cytokines TNFα and IL6, which have been found dysregulated *in vivo* in ALS mouse models and patients^29,43^.

In the culture media of WT-FUS overexpressing cells, the four metabolites (i.e. nitrite -taken as an index of NO production-, PGE_2_, TNFα, and IL6) remained under the detection limit of the specific assays used (see Methods section for details on the assays), as in the media of control cultures (−Dox), suggesting that elevated FUS levels did not change their basal expression (not shown).

To assess whether FUS overexpression changed the reactivity of astrocytes to a typical inflammatory stimulus, the cells were exposed to the pro-inflammatory cytokine IL1β, at the dose of 10 ng/ml for 24 hrs. mRNA expression analyses on cell extracts and metabolite specific assays on culture media were then performed. The dose of IL1β was selected based on the current literature, as the optimal dose to achieve astrocytes activation^44–46^.

**Figure 2:**
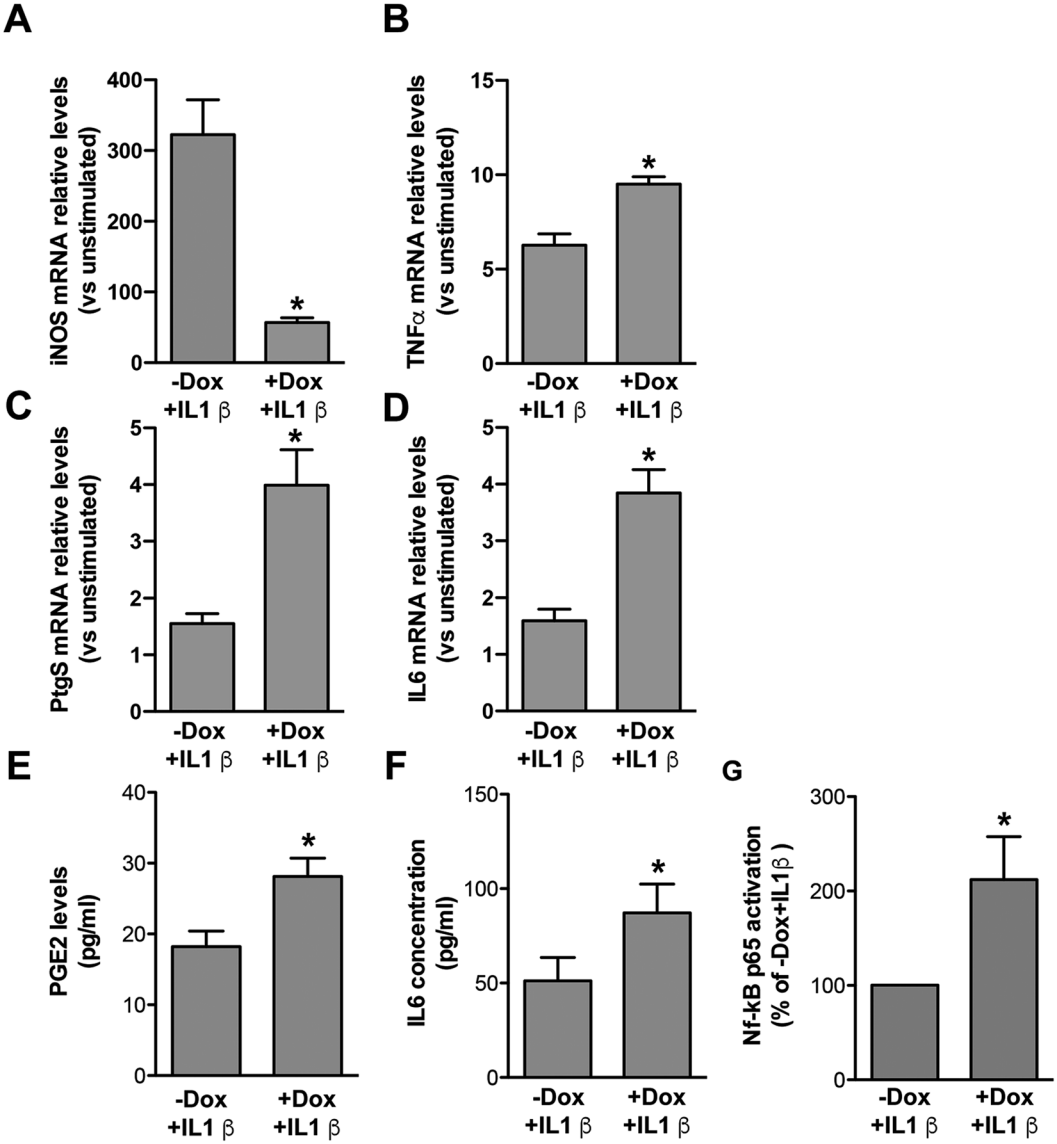
Regulation of inflammatory genes and related proteins/metabolites in IL1β-activated murine WT-FUS overexpressing astrocytes and relative controls, and determination of NF-kB p65 activation. (**A–D**) RT PCR analyses of iNOS (**A**), TNFα (**Β**), PTGS2 (**C**) and IL6 (**D**) mRNA expression upon IL1β stimulation in cultures treated or not with Dox, relative to unstimulated cells (−Dox − IL1β). Data show that TNFα (**Β**), PTGS2 (**C**) and IL6 (**D**) mRNA relative expression upon IL1β stimulation is higher, and that of iNOS (**A**) lower, in cells overexpressing WT-FUS (+Dox + IL1β), compared to non-overexpressing cells (−Dox + IL1β). Data are means ± SEM, *n* = 3–6, *P < 0.05 vs. −Dox + IL1β. (**E**,**F**) PGE_2_ and IL6 were quantified by EIA and ELISA assays, respectively, in the conditioned media collected from astrocyte-like cells differentiated in the presence or in the absence of Dox for 6 days and stimulated in the last 24 hrs with IL1β. In accordance with gene expression data, PGE_2_ and IL6 levels were higher in cells overexpressing WT-FUS (+Dox + IL1β) than in non-overexpressing cells (−Dox + IL1β). Data are means ± SEM, *n* = 3, *P < 0.05 and **P < 0.005 vs. −Dox + IL1β; (**G**) NF-kB p65 activation, assessed by NFkB p65 ELISA-based kit on whole cell lysates, was increased in astrocyte-like cells overexpressing WT-FUS and treated with IL1β for 45 min before collection (+Dox + IL1β). Data are expressed as the percentage of activation respect to control cells (−Dox + IL1β) and are means ± SEM, *n* = 5, *P < 0.05.

As expected, following exposure to IL1β, all transcripts analysed by RT PCR on RNA cell extracts (iNOS, PTGS2, TNFα, and IL6) were upregulated in −Dox cultures (−Dox + IL1β), compared to unstimulated cultures (−Dox − IL1β) (Fig. 2A–D). As shown in panels B–D, their mRNA levels were further upregulated in WT-FUS overexpressing cells (+Dox + IL1β), with the exception of iNOS mRNA (panel A), whose induction was lower than in non-overexpressing cells (−Dox + IL1β).

Notably, a control RFP inducible cell line treated with Dox (+Dox + IL1β) (Supplemental Fig. 3), showed the same response to IL1β than non-induced cells (−Dox + IL1β), confirming the specific effect of FUS overexpression on gene regulation described in Fig. 2.

In accordance with mRNA data, PGE_2_ levels in the culture media of WT-FUS overexpressing cells (+Dox) were higher than in −Dox cultures (Fig. 2E), as assessed by a specific EIA assay.

ELISA analysis showed that IL1β stimulation also caused IL6 increase in the culture media of +Dox cells with respect to −Dox cultures (Fig. 2F), in accordance with the upregulation of the mRNA levels of the cytokine.

As concerns TNFα and nitrite, their levels in culture media were near the limit of detection in all conditions, irrespectively from *FUS* induction and IL1β stimulation (not shown).

To deepen the analysis of astrocyte reactivity to IL1β upon FUS overexpression, we used the TaqMan array for mouse immune response, which allows simultaneous detection of the expression of 92 target genes from immune system functions that fall into 9 classes: Cell Surface Receptors; Stress Response; Oxidoreductases; Proteases; Transcription Factors; Signal Transduction; Cytokines and Cytokine Receptors; Chemokines and Chemokine Receptors; and Cell Cycle and Protein Kinases.

Inflammatory gene expression was compared between astrocyte-like cells overexpressing WT-FUS (+Dox) and control cells (−Dox), both stimulated with 10 ng/ml ILβ in the last 24 hours of differentiation with BMP-4. We found that that 45% of the genes were unchanged (41 genes), 37% expressed under the limit of detection (34 genes), 14% were upregulated (13 genes) and 4% down regulated (4 genes). Some of the unchanged genes showed relevant changes in their expression, though just missing significance (e.g. *Cd68* 3.4 ± 1, *Cd80* 8.5 ± 2.8, *Sele* 2.5 ± 0.5, *Ly96* 2.2 ± 0.4 fold change vs. −Dox). 18% of the analysed genes were significantly dysregulated (up- or down-regulated). In accordance with data presented in Fig. 2B–D, the upregulated genes included *PTGS2* (also known as COX-2), and the pro-inflammatory cytokines *IL6* and *TFN*α. In addition, we found significant upregulation of the pro-inflammatory cytokines *IL5*, *IL7*, and *IL15*, and of colony stimulating factor 2 (*CSF2*), *CD38*, *FN1*, *H2-Eb*, *Lrp2*. Among the downregulated genes, besides *iNOS* (also known as nitric oxide synthase 2), as already shown in Fig. 2A, we found C-C Motif Chemokine Receptor 2 (*Ccr2*), C-X-C Motif Chemokine Ligand 10 (*Cxcl10*), and *Selp* (Table 1).

**Table 1:** Inflammatory gene profiling of astrocyte-like cells expressing human WT- FUS.

	up regulated (fold change)	down regulated (fold change)
Ccr2		0.27 ± 0.03
Cd38	15 ± 2.7	
Csf2	11.9 ± 2.5	
Cxcl10		0.5 ± 0.05
Fn1	4 ± 0.4	
H2-Eb1	2.5 ± 0.3	
Icos	2.5 ± 0.4	
Il15	3.7 ± 0.5	
Il5	4.4 ± 0.7	
Il6	3.0 ± 0.4	
Il7	2.9 ± 0.4	
Lrp2	4.1 ± 0.6	
Nos2		0.4 ± 0.1
Ptgs2	2.6 ± 0.4	
Selp		0.5 ± 0.1
Tnf	4.5 ± 0.8	
Nfatc4	3.4 ± 0.5	

mNPsc were differentiated into astrocyte cells for six days with or without doxycycline (Dox) and stimulated in the last 24 hrs with 10 ng/ml IL1β to assess the effect of FUS overexpression on astrocyte reactivity.

The TaqMan Array 96-well Mouse Immune Response Plate from Applied Biosystems was used to determine the expression of 92 genes from immune system functions (the complete list is reported at the manufacturer website).

Gene expression levels were normalized for four different genes included in the TaqMan Array, and reported as the fold change respect to the levels in cells that were differentiated in the absence of doxycycline and stimulated with IL1β. Values are mean ± SEM (number of independent experiments n = 3). Each sample of each experiment was obtained by collecting RNA form three parallel cultures. Only genes showing significant regulation, as analysed by unpaired Student’s t-test at P < 0.05 are shown.

It has been shown that FUS can interact with the p65 subunit of the transcription factor NF-kB, crucially involved in the regulation of inflammatory genes in glial cells, and activates NF-kB-mediated transcription in the human embryonic kidney 293-cell line^34^.

On these bases, as a possible mechanistic link for the increased reactivity of FUS overexpressing astrocytes to IL1β, we analysed whether FUS overexpression enhanced NF-kB activation in mNPsc-derived astrocytes.

Cells were differentiated into astrocytes for 6 days as above, and stimulated during last 45 min with 10 ng/ml IL1β (+Dox + IL1β). p65 activation, assessed by an ELISA-based assay, was increased in +Dox + IL1β cells as compared to −Dox + IL1β control cells (Fig. 2G), suggesting that FUS overexpression potentiates the NF-kB activation pathway elicited by IL1β.

### FUS overexpression in human astrocyte-like cells increases the expression of inflammatory genes

We extended our analyses to astrocyte-like cells of human origin to validate the data obtained from mouse NPsc-derived astrocytes and strengthen their relevance. We generated a human NP line (hNPsc), isolated from fetal spinal cord, and modified the cells to overexpress WT-FUS under Dox control.

hNPsc displayed mainly bipolar or multipolar morphology (Supplemental Fig. 4A) and under expansion conditions expressed typical neural markers, such as Sox2 and Nestin (Supplemental Fig. 4B,C, respectively), while the expression of GFAP was undetectable in the majority of the cells (Supplemental Fig. 4D). Growth factor removal and administration of BMP-4 induced the expression of the astrocyte marker GFAP in about 70% of cells at 7 days (Supplemental Fig. 4E). hNPsc were also differentiated by removing growth factors; under this culture conditions around 20% of the cells showed neuronal morphology and expressed the neuronal marker MAP2 after 7 days (data not shown). Altogether, these data demonstrate that hNPsc are multipotent.

FUS levels increased under both proliferating or differentiating conditions upon Dox administration (+Dox) compared to control cells (−Dox), as shown in Supplemental Fig. 5.

**Figure 3:**
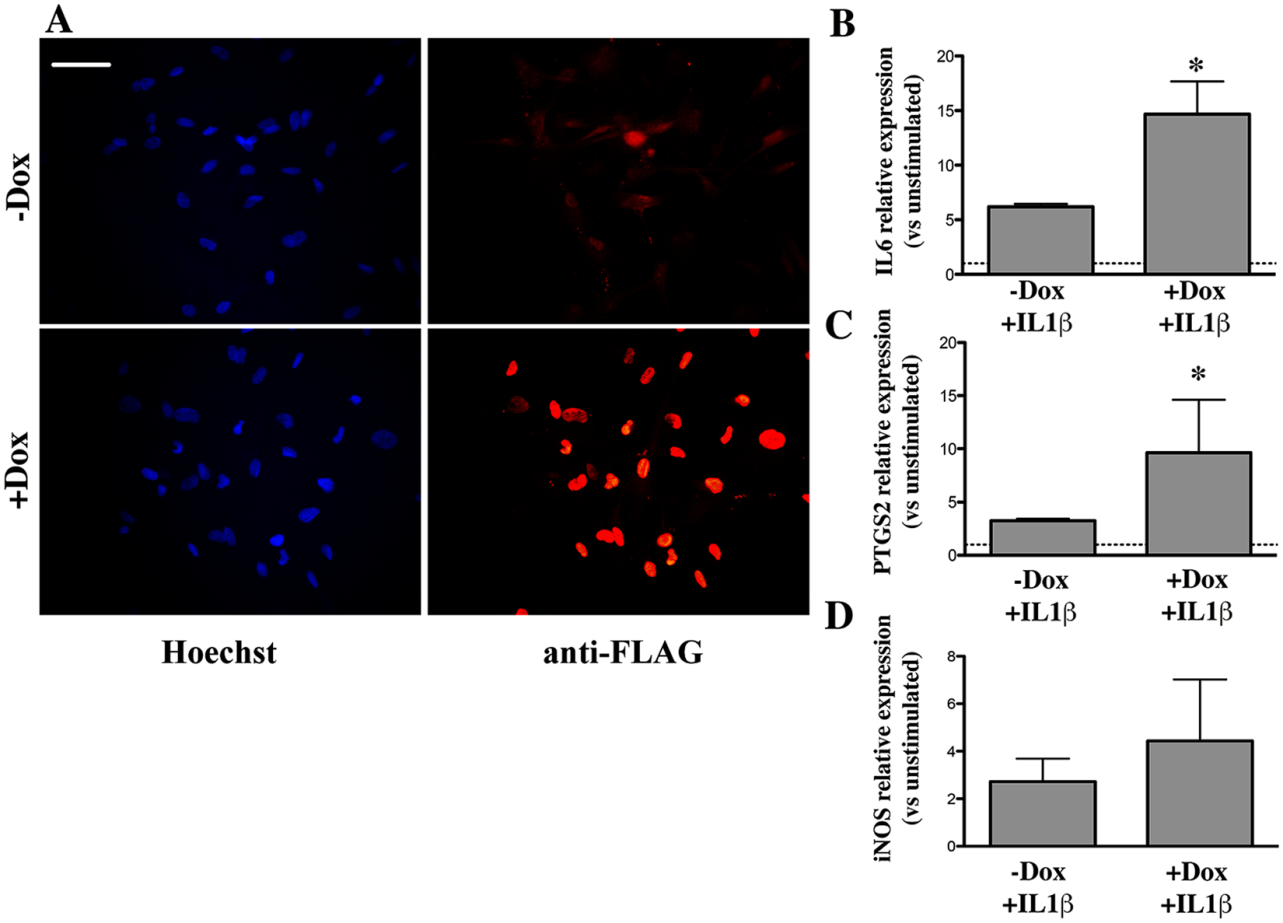
Expression of selected inflammatory genes in human NP-derived astrocytes. hNPsc were differentiated for six days with BMP-4, in the presence or in the absence of doxycycline (Dox) and stimulated in the last 24 hrs with IL1β. (**A**) Example of cultures processed for immunofluorescence and stained with an antibody directed versus the FLAG sequence of human WT-FUS (red) as a control of the efficient induction of transgene expression; in blue are Hoechst-stained nuclei. Scale bar 30 μm. (**B–D**) IL6, PTGS2 and iNOS mRNA relative levels were determined by RT-PCR in cells overexpressing FUS (+Dox + IL1β) or with normal levels of FUS (−Dox + IL1β). Data are expressed as fold change in gene expression, normalized to an endogenous gene (β-actin), and relative to cells that were not stimulated with IL1β (−Dox − IL1β), taken as 1. Data are means ± SEM, n = 3, *P < 0.05.

To assess the effects of FUS overexpression on the reactivity of hNPsc-derived astrocytes to IL1β, cultures were treated with the cytokine for 24 hrs and the mRNA levels of IL6, PTGS2, and iNOS were then analysed by RT-PCR. Representative images of IL1β treated cultures are shown in Fig. 3A. RT-PCR analysis demonstrated that, following 24 hrs of IL1β stimulation, the mRNA levels of IL6 and PTGS2 were higher in FUS-overexpressing hNPsc-derived astrocytes when treated with Dox (+Dox) as compared to untreated cells (−Dox) (Fig. 3B,C). These data are suggesting that, similar to what is observed in mouse cells, increased FUS levels enhance astrocyte reactivity. As for iNOS, its mRNA levels were not significantly modified between +Dox and −Dox conditions (Fig. 3D), most likely reflecting the different regulation of this gene in human cells as compared to mouse cells^47^.

### Conditioned medium from mouse astrocyte-like cells overexpressing WT-FUS reduces neuronal cell number and increases cell death

To assess whether FUS overexpressing astrocytes, showing enhanced production of inflammatory potentially neurotoxic molecules in response to IL1β, can damage neurons we assessed the effects of their conditioned media (CMs) on differentiated cultures.

**Figure 4:**
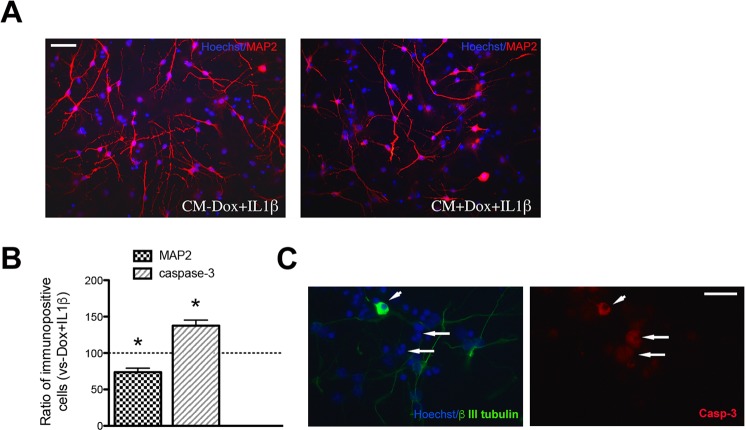
Effects of conditioned media obtained from mouse NP-derived astrocyte-like cells on neuronal cell number and cell death. Non transgenic mNPsc were pre-differentiated for 10 days and then exposed for additional five days to CMs obtained from astrocyte-like cells (CM + Dox + IL1β or CM − Dox + IL1β). (**A**) Representative microphotographic fields showing neuronal cells stained with an anti-MAP2 antibody. Scale bar 30 μm. (**B**) Neuronal and activated Caspase-3 positive cell quantification. The percentage of MAP2 positive neuronal cells was reduced in cultures treated with CM + Dox + IL1β compared with CM − Dox + IL1β treated cultures; data are means ± SEM, *n* = 3, *P < 0.005 (paired Student’s T test). Immunocytochemical detection of activated Caspase-3 showed that CM + Dox + IL1β increased cell death compared to CM − Dox + IL1β after three days of treatment. Quantification of Caspase-3 stained cells is given in the bar graph, and expressed with respect to CM − Dox + IL1β; data are means ± SEM, *n* = 3, *P < 0.0005. (**C**) Representative image showing cells immunostained for βIII tubulin (green) and Casp-3 (red). The arrowhead indicates a neuronal cell expressing activated Casp-3. Note also the presence of Casp-3 positive cells not expressing βIII tubulin (arrows). Scale bar 30 μm).

Non-transgenic mNPsc were first differentiated for 10 days under culture conditions effective for the generation of neuronal cells, as previously described by us and others^39,48^; see also Materials and Methods). Differentiated cultures were then shifted in CMs obtained from 6 days differentiated astrocytes, grown in the presence (CM + Dox) or in the absence of Dox (CM − Dox) and stimulated with 10 ng/ml IL1β in the last 24 hrs (see Materials and Methods for details). As shown in Fig. 4, CM + Dox + IL1β treated cultures showed a significant reduction in the percentage of MAP2 (Fig. 4A,B) and an increase of activated Caspase-3 positive cells (Fig. 4B) as compared to CM − Dox + IL1β condition. To directly assess whether Caspase-3 activation was occurring in neuronal cells, CM-treated cultures were double stained for the neuronal marker βIII tubulin and Caspase-3 (Fig. 4C). Similarly to what observed for MAP2, the percentage of βIII tubulin positive cells was decreased in CM + Dox + IL1β compared with CM − Dox + IL1β treated cultures (17.7 ± 2.5% and 26.2 ± 6.2% of total cell number, respectively). Notably, some of the cells immunopositive for βIII tubulin, displaying severely impaired morphology, were also stained for Caspase-3 (Fig. 4C), suggesting that apoptotic cell death can account for neuronal reduction in CM + Dox + IL1β treated cultures. However, few non-neuronal cells were also Caspase-3 positive, suggesting that toxicity is not limited to neuronal cells but might also affect progenitors and/or other differentiated cells present in the culture.

Cultures treated with CMs from RFP cell line-derived astrocytes exposed to Dox and IL1β showed a percentage of neuronal positive cells (29.5 ± 2.3%) comparable to cultures treated with CMs from FUS-WT-derived astrocytes that had not been exposed to Dox, further confirming that, at least at the dose used, doxycycline does not interfere with astrocyte properties.

Overall, these data suggest that astrocytes overexpressing WT-FUS acquire a cytotoxic functional phenotype when exposed to a typical pro-inflammatory stimulus.

### CMs from mouse astrocytes overexpressing WT-FUS increase the expression of pro-inflammatory markers in microglia

Reactive gliosis in the areas of motor neuron loss and inclusion pathology, in humans and in animal models, implicates cross-talk between astrocytes and microglia and their potential involvement in ALS progression and/or spreading. Therefore, we studied the effect of WT-FUS overexpression in astrocytes in relation to their cross-talk with microglia.

**Figure 5:**
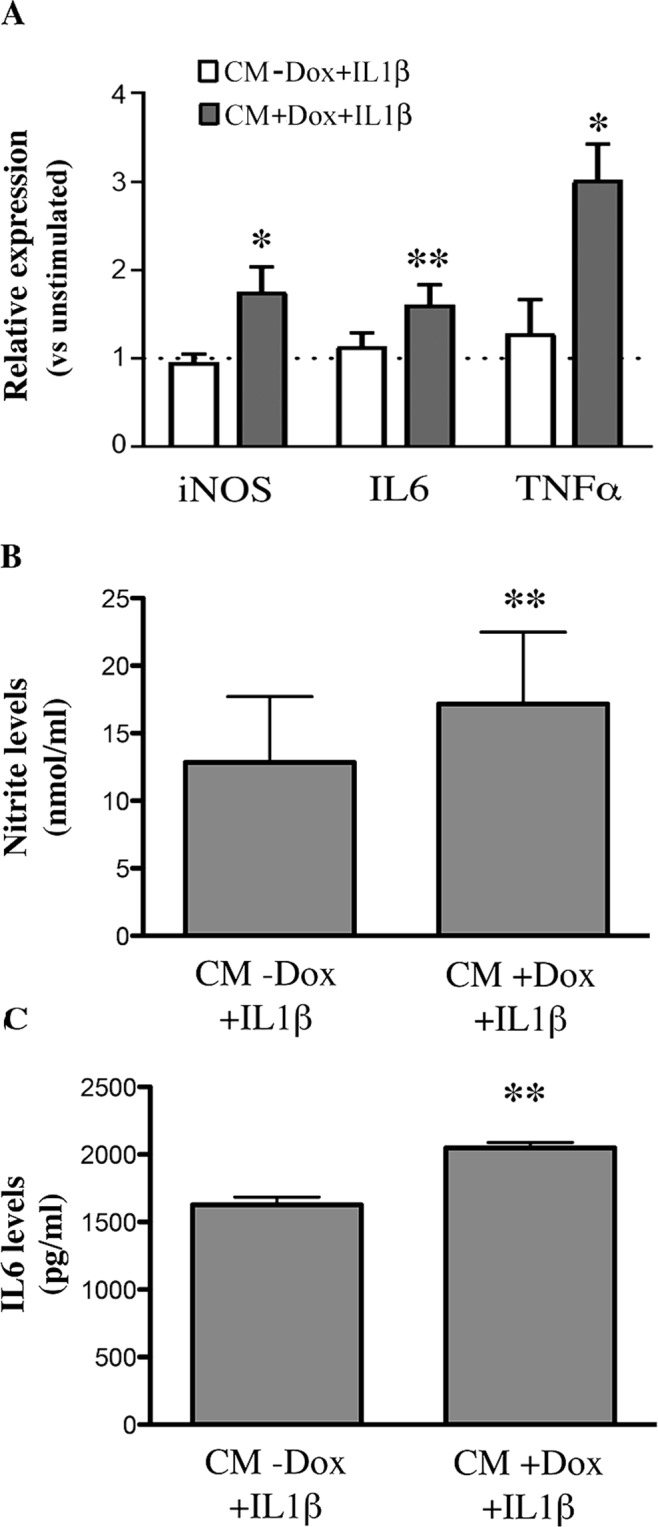
Upregulation of pro-inflammatory genes in microglia exposed to the CMs from transgenic mNPsc-derived astrocytes stimulated with IL1β. (**A**) RT-PCR analysis of the relative mRNA levels of iNOS, IL6 and TNFα, in microglial cultures incubated for 24 hrs with the CMs of astrocyte-like cells, overexpressing FUS and stimulated with 10 ng/ml IL1β (CM + Dox + IL1β) or the CMs from non-overexpressing cells (CM − Dox + IL1β). Data, analyzed by the 2^−ΔΔCt^ method using HPRT as the normalizing gene, are expressed as fold change vs unstimulated microglia, and are the means ± SEM, *n* = 4; *P < 0.05, **P < 0.005 for CM + Dox + IL1β vs CM − Dox + IL1β; paired Student’s T test. (**B**,**C**) Nitrite and IL6 levels accumulated after 24 hrs in the medium of microglia treated with the CMs of astrocyte-like cells overexpressing or not FUS, and activated with IL1β as above (CM + Dox + IL1β and CM − Dox + IL1β). The CMs from FUS transgenic astrocytes induced higher release of both metabolites by microglia, consistently with the mRNA data. Data are the means ± SEM, *n* = 6; **P < 0.005.

Primary microglial cultures were treated with CMs obtained from mNPsc-derived astrocytes, grown with or without Dox and then stimulated with IL1β for 24 hrs (CM + Dox + IL1β and CM − Dox + IL1β, respectively). By RT-PCR analysis, we found that the expression of the pro-inflammatory genes *IL6*, *iNOS*, and *TNF*α were higher in CM + Dox + IL1β treated microglia compared with either unstimulated cultures or cultures exposed to CMs from −Dox + IL1β astrocytes (Fig. 5A).

In accordance with gene expression data, we found that nitrites and IL6 released from microglia treated with CM + Dox + IL1β were significantly higher as compared to CM − Dox + IL1β treated cultures (Fig. 5B,C).

As described above, nitrite levels in astrocyte CMs were below the detection limit of the assay in all conditions; we can thus conclude that the levels measured in microglial media were produced by microglia itself.

As for IL-6, the Elisa kit used for detection of the cytokine in the culture media of rat microglia was specific for rat, and it is expected not to cross-react with IL6 from mouse astrocytes. In addition, the levels of the cytokine measured in mouse astrocyte CMs (see Fig. 2F) were far below the levels measured in rat microglial media (Fig. 5C). For these reasons, we can state that the levels shown in Fig. 5C are related, at least mostly, to IL6 released by microglia.

Altogether, these data indicate that the overexpression of WT-FUS in mNPsc-derived astrocytes alters their cross-talk with microglia, upon IL1β-mediated activation, and determines the acquisition of a pro-inflammatory profile by microglia.

**Figure 6:**
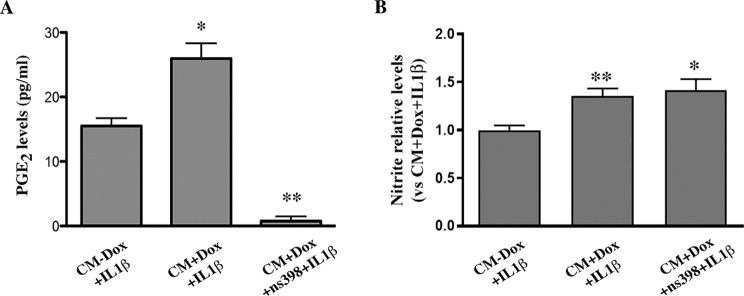
PTGS2 inhibition by NS-398 in transgenic astrocyte-like cells does not alter their capability to stimulate microglial activation. (**A**) mNPsc-derived astrocytes overexpressing FUS and treated for 24 hrs with 10 ng/ml IL1β in the presence of the selective PTGS2 inhibitor NS-398 (10 μM), produced negligible amounts of PGE_2_, compared to non-inhibited cells, as assessed by EIA analysis. Data are means ± SEM, *n* = 3; *P < 0.05. (**B**) Nitrite levels in the medium of microglia exposed to CMs from FUS overexpressing cells treated with 10 ng/ml IL1β in the presence of NS-398 (CM + Dox + IL1β + NS-398) were comparable to the levels induced by the CMs from non-inhibited astrocytes (CM + Dox + IL1β). Data are expressed as the fold increase versus nitrite levels produced by microglia exposed to CMs from non transgenic astrocytes (CM − Dox + IL1β), and are means ± SEM, *n* = 3.

Previous studies have shown that prostanoid-mediated signaling controls microglial activation, and its inhibition reduces motor neuron loss in mouse models of ALS^49–51^. In view of the upregulation of PTGS2 found in WT-FUS overexpressing astrocyte-like cells stimulated with IL1β, we addressed whether PTGS2-derived prostanoids could be responsible for the enhanced reactivity of microglia exposed to their CMs. To this aim, we used the selective PTGS2 inhibitor, NS-398 at the concentration of 10 μM, to inhibit PTGS2 activity in WT-FUS overexpressing astrocytes. As expected, following NS-398 treatment, PGE_2_ levels accumulated in the CMs, as an index of PTGS2 activity, dropped under the detection limit of the EIA (Fig. 6A). Microglial cultures exposed to the CMs from NS-398 inhibited FUS-overexpressing astrocytes, produced levels of nitrite comparable to those of microglia exposed to CMs from not-inhibited astrocytes, indicating that microglia activation following exposure to CMs from FUS-overexpressing astrocytes was not dependent on increased prostanoids in their CMs (Fig. 6B).

## Discussion

Glial contribution to MN degeneration is increasingly recognized in ALS-models and patients^22,27,52^; however, it is still not well established whether changes in WT-FUS levels due to mutations in the 3′UTR, as found in a subset of ALS patients, can affect glial properties and eventually sustain neuron degeneration. Although several studies indicate that high levels of wild-type FUS are toxic for MNs^13–16^, and MNn degeneration was demonstrated to be at least in part dependent on toxic gain of function in mouse models of ALS-FUS^17,18^, astrogliosis and microgliosis were also found in these same mice, as in SOD1 mice and ALS patients, suggesting that non-cell autonomous mechanisms can take place in FUS-mediated pathology, and contribute to disease progression. Our data clearly speak in favor of this possibility.

Here we showed that overexpression of WT-FUS in spinal cord astrocytes altered their reactivity to a pro-inflammatory stimulus, modified their cross-talk with microglia, and caused non-cell autonomous cell death.

FUS levels achieved in our cell model are comparable to those found in FUS-ALS mice overexpressing human WT-FUS^16^, supporting that our study can be informative of pathological mechanisms sustained by FUS overexpression.

We found that in spinal cord NPs-derived astrocytes engineered to overexpress conditionally the wild-type sequence of the protein, FUS was mainly localized within nuclei, and only a very small fraction of the cells was faintly stained in the cytoplasm. Protein delocalization and intracytoplasmic inclusions have been found in FUS-associated ALS, either in neurons or glia^4^, and their formation was proposed as a mechanism driving neuronal degeneration. Our findings, in agreement with other studies showing that elevated WT-FUS remains predominantly in the nucleus^11,18,43^, demonstrate that deregulation of FUS expression in astrocytes, even in the absence of overt delocalization, can drive a relevant rearrangement of their inflammatory and neurotoxic properties.

The nuclear elevation of WT-FUS altered astrocyte reactivity, as revealed by the dysregulation of a panel of inflammatory genes in FUS overexpressing cells, compared to non-overexpressing astrocytes, in the presence of IL1β, used to mimic an inflammatory environment. In particular, the higher mRNA expression of pro-inflammatory cytokines (IL5-6-7-15, TFNα, CSF2), the inducible inflammatory enzyme PTGS2, adhesion molecules (FN1, ICAM), and the multi-ligand endocytic receptor LRP2, indicates the acquisition of a more reactive phenotype compared to non-overexpressing cells. On the other hand, the reduced expression of Ccr2, CxcL10, and iNOS, in activated FUS-overexpressing astrocytes (Table 1), may indicate an incomplete/dysregulated acquisition of inflammatory functions in overexpressing astrocytes.

A variety of pro-inflammatory cytokines and growth factors, including IL-5 and IL-15, have previously been reported to be elevated in cerebrospinal fluid of patients with ALS^53^ and their abnormal expression has been associated with ALS clinical status^54^. PTGS2, IL6, and TNFα were similarly found dysregulated in ALS mouse models and patients, further supporting the relevance of our findings.

Consistently with the enhanced mRNA expression of PTGS2 and IL6 in FUS overexpressing astrocytes, PGE_2_ and IL6 protein levels were increased in their conditioned medium. TNFα, although increased at the mRNA level, was undetectable in the medium. In a recent study, analyzing the effects of the overexpression of FUSR521G mutation in primary astrocytes, Kia and colleagues^43^ found increased release of TNFα in the medium, and identified TNFα as a mediator of motor neuron toxicity. The lack of TNFα accumulation in the condition medium of the WT-FUS overexpressing astrocytes used in our study is likely reflecting different consequences of the overexpression of the mutated *vs*. the WT form of the protein. Alternatively, it could reflect a different maturational state between primary astrocytes used by^43^ and the astrocyte-like cells used in our study, or even regional differences in the areas of origin of these two astrocyte populations.

Our results extend previous observations and further establish that astrocytes with aberrant FUS expression can contribute to neuronal death upon activation with IL1β. Indeed, their conditioned medium led to a reduction in the percentage of neuronal cells, as evaluated by MAP2 and βIII tubulin stainings and an increase in activated Caspase-3 (Casp-3) stained cells. The identification of double labeled cells (Casp-3+/βIII tubulin+) indicates that at least part of the neuronal cells were dying through the activation of the apoptotic pathway. Several Casp-3 positive cells were βIII tubulin negative, suggesting that cell death was also affecting other cell types in culture, such as neuronal progenitors and/or glial cells.

Among the multitude of factors into the CMs that could be responsible of cell toxicity, pro-inflammatory cytokines (e.g. IL6), found upregulated in FUS overexpressing astrocytes are potential good candidates, as suggested by *in vitro* and *in vivo* models of different neuropathologies^55,56^. A possible dysregulation of the expression of neuroprotective factors could also contribute to the effect observed. The specific factors responsible for neurotoxicity were however not identified in the present study.

The striking functional and structural similarities between FUS and TAR DNA-binding protein-43 (TDP-43), which are both DNA/RNA binding proteins whose mutations have been linked to the pathogenesis of both ALS and frontotemporal dementia (FDT), suggest that abnormal functioning/expression of these two related proteins can be pivotal event in ALS and FTD^8^.

Interestingly, Tong and coworkers, previously showed that, similarly to what we observed with WT-FUS overexpression, an increase in the expression of normal TDP-43 in astrocytes is sufficient to cause activation of astrocytes themselves and microglia, and non-cell-autonomous death of motor neurons^57^. In the same model, it was shown that motor neuron death likely involved deficiency in neuroprotective genes and induction of neurotoxic genes in astrocytes.

In addition, our data demonstrate that co-stimulatory molecules, such as CD38 and Icos, potently associated with T-cell activation and correlated with astrocyte ability to stimulate pro-inflammatory and immune regulatory patterns of cytokine production, were more upregulated in WT-FUS overexpressing astrocytes than in control cells. The co-stimulatory molecule CD80 was also induced in WT-FUS overexpressing cells, with an effect that just missed significance due to variability in the levels of induction among different cultures. Altogether, these observations suggest that WT-FUS overexpression may profoundly modify the cross talk of astrocytes with T-cells.

A global dysregulation of T-cell functions and immune changes have been related to an increased disease progression, decreased survival as well as production of pro-inflammatory effectors in experimental ALS and patients^26,58^. Interestingly, a recent transcriptomic analysis of motor cortex samples from sporadic ALS patients identified the upregulation of membrane protein-encoding genes involved in T-cell activation and proliferation (including ICOS and ICOS-L) in a subgroup of patients characterized by increased expression of genes involved in the inflammatory response^59^. From this perspective, the enhanced expression of costimulatory molecules found in FUS overexpressing astrocytes could be part of a pathogenic mechanism sustained by alterations of FUS levels.

The molecular mechanism underlying the enhanced expression of inflammatory and costimulatory genes in FUS overexpressing astrocytes requires further elucidations. However, our data indicate that the p65 subunit of NF-kB, a transcription factor crucially involved in the transcription of these genes and a master regulator of inflammation^60^, was more activated in murine FUS overexpressing astrocytes than in control cells. The similar upregulation of Nf-kB target genes in murine and human derived astrocytes overexpressing WT-FUS may suggest a common mechanism of NF-kB activation dependent on FUS in both cell types. Our results are consistent with previous data on the human embryonic kidney 293-cell line, in which FUS was shown to mediate transcription of NF-kB-dependent genes and to interact with p65^7,34^. Similarly, in other tumor cell lines, the FUS-DDIT3 fusion oncogene was found to regulate NF-kB target genes^61^. FUS deregulation can thus elicit and/or contribute to the neuroinflammatory response in part by enhancing NF-kB activation, a mechanism also proposed for TDP-43 in ALS^32^. NF-kB and kinase signaling pathways were also found activated in familial and sporadic ALS astrocytes^22^.

A recent study revealed that the timing of astrocytic NF-kB activation drives a stage-specific neuroimmunological response in the SOD1 (G93A) ALS model: NF-kB activation promotes the expression of molecules that once released amplify microglial proliferation and protective functions in the presymptomatic phase of ALS, but also promotes detrimental microglial functions in the symptomatic phase^62^.

Besides astrocytes, microglial cells are increasingly recognized as crucial players in ALS pathology^63^, and microglial activation has been described in ALS patients and in animal models of ALS, including rodents carrying mutations of *FUS*^16^. As also suggested by the Ouali Alami *et al*. study^62^, the altered interaction of microglia with other immune cells^26^, and neurons themselves can profoundly affect the pathogenic process and its progression.

Notably, our study demonstrates that altered FUS expression in activated astrocytes alters their communication with microglia, as FUS-overexpressing astrocytes were able to induce activation of primary microglia more efficiently than control cells. Indeed, microglia exposed to astrocyte CMs upregulated the expression of typical pro-inflammatory mediators such as IL6, iNOS, and TNFα, suggesting a mechanism of amplification of the inflammatory response mediated by FUS, with the enhanced release of potentially harmful and neurotoxic molecules.

Pre-treatment of FUS overexpressing astrocytes with the selective PTGS2 inhibitor NS-398, did not halt the activation of microglia in response to astrocyte CMs, suggesting that under these conditions, other molecules than prostanoids into the CMs were major players in modulating microglia activation. Among those, the pro-inflammatory cytokines IL6, IL5, IL7, IL15, found upregulated in FUS overexpressing astrocytes, are likely candidates as drivers of microglial activation by CMs, as well as other molecules possibly under astrocytic NF-kB transcriptional control.

Overall, our study adds further insights into the pathways modulated by FUS in glial cells and shows that elevated levels of the protein, mainly in the nucleus, can shape astrocyte properties, steering them toward a functional phenotype that can affect neuronal survival and promote microglial activation, thus triggering a possible amplifying neurotoxic loop. As several mutations affecting FUS coding sequence cause protein delocalization with formation of intracytoplasmic inclusions and reduction in FUS nuclear levels, both gain of function in the cytoplasm and loss of function in the nucleus, have been proposed to participate into the sequential cascade culminating into pathology^64^.

Studies from mouse models of FUS pathology, describing neurodegeneration in the absence of cytoplasmic pathology or significant mislocalization of FUS, strongly suggest that also nuclear gain of toxic function by mutant FUS may represent an important pathological mechanism^65–67^.

In the same line, our data show that retention/accumulation of aberrant levels of FUS into the nucleus can profoundly impair glial functions, likely by interfering with normal alternative splicing and/or transcription of target genes. A better understanding of the complex mechanisms underlying the control of glial functions and cross-talk with other cell types by FUS will contribute to identify common or unique cellular pathways involved in MN degeneration in different forms of ALS.

## Material and Methods

### Cell cultures

#### Expansion and differentiation of mouse and human neural progenitor cells

Mouse neural progenitor cells were previously derived from the spinal cord (mNPsc) of male and female CD1 mice at embryonic day E13.5, and grown as described in^68–71^.

Briefly, mNPsc were grown on 10 μg/ml poly-L-ornithine and 2,5 μg/ml laminin coated flasks in *expansion medium* constituted by DMEM/F12 (Gibco), supplemented with penicillin/streptomycin at 1:100 dilution (P/S; Sigma, Catalog # P4333, formulated as 100X concentration to contain 10,000 units penicillin and 10 mg streptomycin/mL), 1:100 of 100x Glutamax (Gibco, Catalog # 35050061), 1:100 of N2 Supplement (Gibco, Catalog # 17502001, provided as a 100X chemically defined formulation), 10 ng/ml basic fibroblast growth factor (bFGF; Peprotech) and 20 ng/ml epidermal growth factor (EGF; Peprotech).

mNPsc were differentiated into neurons by a previously established protocol. Under this culture condition, around 55% of the cells expressed markers and displayed morphology of neuronal cells^39,72^. Cells were plated at the density of 2 × 10^4^ cells/cm^2^ and 24 hrs later *expansion medium* was replaced with *neuronal differentiation medium* containing DMEM/F12, 1:100 of 100x P/S, 1:100 of 100x Glutamax, 10 ng/ml bFGF, 1:100 of 100x N2S, 1:50 of 50X B27 (Gibco, Catalog # 17504, provided as a 50X serum-free supplement) and 1 μM N-[(3,5-difluorophenyl)acetyl]-lalanyl-2-phenyl]glycine-1,1-dimethylethyl ester (DAPT; Tocris). The whole medium was replaced once during the first 6 days of differentiation. Then cells were differentiated for additional 4 days in fresh medium withdrawn of growth factors and additioned with 20 ng/ml brain derived neurotrophic factor (BDNF; Peprotech).

mNPsc were differentiated into astrocyte-like cells by replacing *expansion medium* with *astrocyte differentiation medium* constituted by DMEM/F12 containing 1:100 of 100x N2S, 1:50 of 50x B27, 1:100 of 100x Glutamax, 1:100 of 100x P/S, and 25 ng/ml mouse bone morphogenetic protein-4 (BMP-4; Peprotech). The whole medium was changed every 3 days with fresh medium. Under these culture conditions, more than 90% of the cells expressed the glial marker GFAP, and neuronal cells were virtually absent (see also^36,39^).

The human neural progenitor cell line was derived from the spinal cord (hNPsc) of a 9 week post-conception fetus. Fetuses were obtained from Lund and Malmö University Hospitals, according to guidelines approved by the Lund/Malmö Ethical Committee. Informed consent for donation of foetal tissue was obtained from the mother.

Specifically, the human line was derived from P0 neurospheres. hNPsc were expanded according to a previously established protocol^73^. Cells were plated on 0.1 μg/ml poly-L-ornithine and 10 μg/ml laminin coated flasks, and grown in *human expansion medium* constituted by DMEM/F12 supplemented with 1:50 of 50x B27 minus vitamin A (Gibco), 1:100 of 100x P/S, 1:100 of 100x Glutamax, 10 ng/ml bFGF (R&D) and 10 ng/ml EGF (R&D).

For hNPsc differentiation, cells were plated at the density of 1 × 10^4^ cells/cm^2^ in *human expansion medium*, and 24 hours later switched in a medium containing 1:100 of 100X N2S, 1:50 of 50x B27 (Gibco), 1:100 of 100x P/S, 1:100 of 100x Glutamax and supplemented with 20 ng/ml human recombinant BDNF, or 25 ng/ml human recombinant BMP-4 (Peprotech).

#### Generation of stably engineered cell lines

For the generation of stable mNPsc lines overexpressing wild-type FUS or the red fluorescent protein (RFP), cells were engineered with the appropriated plasmids, which allowed for the doxycycline inducible expression of the genes of interest^41^.

Plasmids were delivered into the cells by electroporation (Nucleofector; Lonza), according to manufacturer’s instruction (see also^39,69^ for further details) and selected with puromycin (1 μg/ml).

mNPsc-RFP cells were used to determine the effective dose of doxycycline to induce transgene expression. The dose of 50 ng/ml was chosen as the lowest, among the tested doses, able to induce the transgene expression in a very consistent number of cells (about 80–85%), without affecting cell survival, proliferation and expression of selected inflammatory genes (data not shown).

Similar procedures and doses of antibiotics for cell selection and gene induction were used during the generation of stably transfected hNPsc lines.

#### Primary microglial cultures

Primary microglia was obtained from mixed glial cultures prepared from the cerebral cortex of 1-day-old male and female Wistar rats (RRID:RGD_13508588) from Charles River following previously published protocols^74^ and in accordance with the European Communities Council Directive N. 2010/63/EU and the Italian Law Decree n° 26/2014 (authorization from Ministry of Health:152/2016-PR).

In brief, mixed cultures were maintained for 11 days in Basal Eagle’s Medium (BME, Gibco, Catalog # 41010), supplemented with 10% heat-inactivated foetal bovine serum (FBS, ultra-low endotoxin, South American Origin, EuroClone, Catalog # ECS0186L), 2 mM L-glutamine (Gibco, Catalog # 25030081, provided as a 200 mM stock solution) and 100 μg/ml gentamicin (*basal medium*). After this time, microglial cells were harvested by mild shaking, resuspended in *basal medium*, and seeded on uncoated plastic wells at the density of 1 × 10^5^ cell/cm^2^. Cell viability was greater than 95%, as tested by Trypan Blue exclusion. Immunostaining revealed that cultures consisted of 96% positive cells for the microglia/macrophage marker CD68. After 24 hrs in culture, *basal medium* was replaced with the CMs from transgenic mNPsc, and the cells incubated for additional 24 hrs. The media were then collected and stored at −70 °C until subsequent analyses.

### Preparation of conditioned media and RNA

Transgenic mNPsc were maintained in *astrocyte differentiation medium* for 6 days, in the presence or in the absence of 50 ng/ml of doxycycline. Cells were then washed three times with PBS and cultured for the last 24 hrs in a medium constituted by DMEM/F12, 1:100 of 100x P/S, 1:100 of 100x Glutamax, 1:50 of 50x B27, 1:100 of 100x N2S and 10 ng/ml IL1β, without doxyclycline to avoid its potential confounding effects. CMs were collected, centrifuged at 1500 rpm for 5 min and stored at −20 °C. The cells were lysed in Trizol (Sigma-Aldrich) for total RNA extraction and subsequent gene expression analyses (see below).

Thawed CMs were assessed for cytokines, nitrite, and prostaglandin E_2_ (PGE_2_) levels, and for their effects on neuronal or microglia cells, as described below.

### NF-kB p65 activation assay

NF-kB activation was assessed in 6 days differentiated astrocyte-like cells (see above), stimulated, 45 min before being collected, with 10 ng/ml of IL1β in fresh medium.

Proteins were collected by using an extraction kit (Active Motif, Catalog # 40010), according to manufacturer’s instruction. The whole-cell lysates from mNPsc were concentrated by means of Amicon Ultra-0.5 centrifugal filter devices (Millipore) to achieve the recommended concentration of 20 μg of protein per well per sample (20 μl volume). The protein concentration was determined before and after concentration, by the BCA protein assay (Pierce).

NF-kB activation in whole-cell lysates was determined by the TransAM NFkB p65 kit (Active Motif, Catalog # 40096), following the manufacturer’s instructions. This 96 well ELISA-based kit provides colorimetric readout of NF-kB p65 subunit activation, quantified by spectrophotometry at the wavelength of 450 nm (reference wavelength 655 nm). As a positive control for p65 activation, Jurkart nuclear extracts, provided with the kit, were used. Detection limit: <1 μg lysate/well.

### Cytokines, PGE_2_ and NO determination

Specific ELISAs for mTNFα, mIL6 (Thermo Scientific, Catalog # EMTNFA and # EM2IL6, respectively), rIL6 (PicoKine ELISA Kit, Boster Biological Technology, Catalog # EK0412), and high sensitivity enzyme immunoassay (EIA) for PGE_2_ (Assay Designs; Catalog # 931-001) were used to assay cytokines and PGE_2_ levels in CMs from transgenic mNPsc and microglia, following the manufacturer’s instructions. The ranges of determination are: 50–2450 pg/ml for mTNFα, 7.8–500 pg/ml for mIL6; 62.5–4000 pg/ml for rIL6; 7.8–1000 pg/ml for PGE_2_.

The production of nitric oxide (NO) was determined by measuring the content of nitrite, one of the end products of NO oxidation in the media, as previously described^75^. All chemical for the NO assay were from Sigma.

### Immunocytochemistry

Immunocytochemistry was performed on NPsc fixed with 4% paraformaldehyde (PFA) in PBS for 15 min at room temperature. After fixation, cultures were washed with PBS and then incubated in PBS containing 5% of the appropriate normal serum and 0.025% Triton X-100 (preincubation solution) for 1 h at room temperature.

Subsequently, cultures were incubated at 4 °C overnight in the preincubation solution containing the primary antibodies listed below. Cells were washed 3 times with PBS and incubated for 1 h at room temperature in the incubating solution containing 10 μg/mL Hoechst 33342 and the appropriate alexa fluor 488- and/or Cy3-conjugated (Jackson Immunoresearch) secondary antibodies at dilution 1:200. After rinsing with PBS, cells were coverslipped with DAKO mounting medium (Dako).

The following primary antibodies were used: rabbit polyclonal anti-FLAG (1:1000; Sigma, RRID:AB_439687); mouse monoclonal anti-FUS (1:1000; Santa Cruz, RRID:AB_2105208); mouse monoclonal anti-SOX-2 (1:50; R&D Systems, RRID:AB_358009); mouse monoclonal anti-nestin (1:200; Millipore, RRID:AB_11211837); monoclonal anti-beta III tubulin (1:500; Promega, RRID:AB_430874); mouse monoclonal anti-S100b (1:100; Abcam, RRID:AB_1142710); rabbit polyclonal anti-MAP-2 (1:500; Millipore, RRID:AB_91939); rabbit polyclonal anti-Caspase 3 (1:20; Millipore, RRID:AB_91556); rabbit polyclonal anti-GFAP (1:200; Cell signaling Technology, RRID:AB_2631098); mouse monoclonal anti βIII tubulin (1:500; Promega, RRID:AB_430874). Validation data for the antibodies are available from the companies.

The percentages of immunopositive cells for differentiation or apoptotic markers (such as Caspase-3), in the different experimental conditions, were determined by counting immunolabeled cells in at least 10–15 microscopy randomly chosen fields of 3 different microscope slides for each condition, in at least 3 independent experiments. The samples were large enough to minimize the statistical impact of rare outliers in each group. The investigator in charge of counting was blinded to experimental groups. Groups were identified by letters, and the code was disclosed at the end of the analyses.

### Western blotting

Cells were lysed and harvested in RIPA buffer (320 mM Sucrose, 50 mM TRIS pH 7.5, 1% Tryton, 10% glycerol and 1% of inhibitor of proteases cocktail (Sigma-Aldrich), incubated on ice for 30 min and centrifuged for 12 min at 13000 g. Protein amounts were quantified by Bradford assay (Bio-Rad) and 10 μg of proteins were subjected to SDS-polyacrylamide gel electrophoresis (4% and 10% for stacking and running gel, respectively) transferred overnight onto polyvinylidene fluoride membrane (Immobilion-PSQ, Millipore). Membranes were blocked in 5% non-fat dry milk and then incubated with the following primary antibodies: monoclonal anti-FUS (1:700, Santa Cruz) and anti-beta-Actin (1:10000, Sigma-Aldrich, RRID:AB_476744). Blots were washed twice, incubated with the appropriate horseradish peroxidase secondary antibodies for 2 hrs (1:10000, Jackson Immunoresearch), and after three additional washing steps, incubated in lumi-light enhanced chemiluminescence substrate (Bio-Rad) and exposed into Chemidoc (Bio-Rad). Densitometric analyses on scanned blots were performed using the ImageLab program (Bio-Rad). Row western blotting images are provided as supporting document.

### Quantitative real-time polymerase chain reaction (RT-PCR) analysis

Total RNA was extracted with the Trizol extraction procedure (Invitrogen, Carlsbad, CA); to remove any traces of genomic DNA, total RNA was treated with DNAse I (Ambion, Grand Island, NY). For each RNA sample, 1 μg total RNA was reverse transcribed with random hexamers (Promega) and MMLV reverse transcriptase (Promega)^71^.

For inflammatory gene expression in mNPsc-derived astrocytes, overexpressing or not WT-FUS in the presence of IL1β, we used the TaqMan Array 96-well Mouse Immune Response Plate (Catalog # 4414079). Gene expression was performed by using TaqMan Universal Master mix II (Applied Biosystem) on a 7500 RT-PCR (Applied Biosystems). The values obtained were normalized for the four different genes (18S, Gapdh, Gusb, Hprt1) included in the TaqMan Array plate and expressed as fold change over non-overexpressing cells.

For RT-PCR analyses of selected genes in mNPsc- and hNPsc-derived astrocytes and in rat microglia, we used the following primer pairs:

**Primer sequences (mouse):**

*mβ–actin: For GCGCAAGTACTCTGTGGA Rev AAGGGTGTAAACGCAGCT*

*mGFAP: For CGAAGAAAACCGCATCACCAT Rev GGCCTTCTGACACGGATTTG*

*mS100β: For TGCCCTCATTGATGTCTTCCA Rev GAGAGAGCTCGTTGTTGATAA*

*mAquaporin 4: For GAGTCACCACGGTTCATGGA Rev CGTTTGGAATCACAGCTGGC*

*mNestin: For TGCCGAAGAGCTGGAGAG Rev CTCGCAGAGCCTCTAACTCG*

*mEtv5: For GGGAGAGACAAAAACCACCA Rev GCGCTAACCCTGAACACACT*

*mFGFR3: For CTTCAGTGTGCGTGTAAC Rev CGTTTGGAATCACAGCTGGC*

*mTNFα: For CATCTTCTCAAAATTCGAGTGACAA Rev TGGGAGTAGACAAGGTACAACCC*

*mIL6: For GAGGATACCACTCCCAACAGA Rev AAGTGCATCATCGTTGTTCATACA*

*miNOS: For TCTTCGGTGCAGTCTTTTCC Rev GTGCCAGAAGCTGGAACTCT*

**Primer sequences (human):**

*hβ–actin: Qiagen*

*hFUS: For GCTGCCATCACAAGCATAGC Rev CAGCCTGGATGACAGAGCAA*

*hIL6: For CTCAGCCCTGAGAAAGGAGA Rev TTTCAGCCATCTTTGGAAGG*

*hPTGS2: For CGCTCAGCCATACAGCAAAT Rev CCGGGTACAATCGCACTTAT*

*hiNOS: For ACAAGCCTACCCCTCCAGAT Rev TCCCGTCAGTTGGTAGGTTC*

**Primer sequences (rat):**

*rHPRT: For CTCATGGACTGATTATGGACAGGAC Rev GCAGGTCAGCAAAGAACTTATAGCC*

*rIL6: For TCCTACCCCAACTTCCAATGCTC Rev TTGGATGGTCTTGGTCCTTAGCC*

*rTNFα: For AAATGGGCTCCCTCTCATCAGTTC Rev TCTGCTTGGTGGTTTGCTACGAC*

*riNOS: For GCC ACC TCG GAT ATC TCT TG Rev TCT GGG TCC TCT GGT CAA AC*

Annealing temperature was 60 °C for all the primer pairs listed. All samples were run in triplicate, and each well of PCR contained 20 μl as a final volume of reaction, including 2 μl of cDNA corresponding to 20 ng of total RNA, 0.75 μM of each primer and 10 μl of PCR master mix. Thermal cycling conditions were as follow: 1 cycle at 95 °C for 10 min; 40 cycles 95 °C for 15 sec and 60 °C for 1 min.

Expression levels of genes of interest were compared between culture conditions using the Relative Quantification (ΔΔCt) Study of Applied Biosystems 7500 System SDS Software. HPRT or β-actin were used as internal control genes and amplification specificity was checked using a melting curve, following the manufacturer’s instructions.

### Statistical analysis

Data are expressed as mean ± SEM of at least three independent experiments (run in triplicates). Exact numbers (n) for all the experiments are provided in the figure legends.

The experimental design and the statistical analysis of the data in this study followed the methodology and the standards usually used in explorative *in vitro* studies. In the experimental models adopted, external sources of variability are controlled, and residual variability arises from random error of replicates, as also demonstrated by the low SEM. On these bases, we adopted two-sided Student’s t-test as the more powerful test to evidence possible differences between two groups. GraphPad Prism software was used. P values ≤ 0.05 were considered as significant.

## Supplementary information

Supplementary figures
