# Supplementary material for: Increased FUS levels in astrocytes leads to astrocyte and microglia activation and neuronal death
Source: Sci Rep. 2019 Mar 14;9:4572. doi: 10.1038/s41598-019-41040-4 (PMC6418113; doi:10.1038/s41598-019-41040-4)
Supplement: Supplementary file 1 — Supplementary figures [file 41598_2019_41040_MOESM1_ESM.pdf]

Increased FUS levels in astrocytes leads to astrocyte and microglia activation and neuronal death

Maria Antonietta Ajmone Cat ,Angela Onori, Camilla Toselli, Eleonora Stronati, Mariangela Morlando, Irene Bozzoni, Emanuela Monni, Zaal Kokaia, Giuseppe Lupo, Luisa Minghetti, Stefano Biagioni, Emanuele Cacci

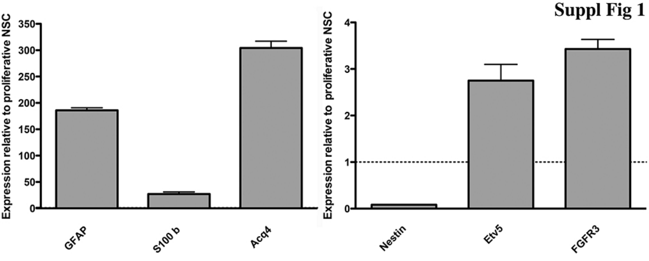

Suppl. Figure 1.
Expression of astrocytic markers in mNPsc differentiated in the presence of BMP-4. RT-PCR analysis shows increased expression of genes typically upregulated during astrocyte differentiation (GFAP, S100β, Aquaporin4, FGF3, Etv5). Note that the neural marker nestin is downregulated in mNPsc differentiating cells. Relative mRNA levels were assessed by using the 2-ΔΔCt method. Data are expressed as the fold change in gene expression normalized to the endogenous gene (β-actin) and relative to proliferating cells (n = 3, in triplicate).

Suppl. Fig. 2

Increased FUS levels in astrocytes leads to astrocyte and microglia activation and neuronal death

Maria Antonietta Ajmone Cat ,Angela Onori, Camilla Toselli, Eleonora Stronati, Mariangela Morlando, Irene Bozzoni, Emanuela Monni, Zaal Kokaia, Giuseppe Lupo, Luisa Minghetti, Stefano Biagioni, Emanuele Cacci

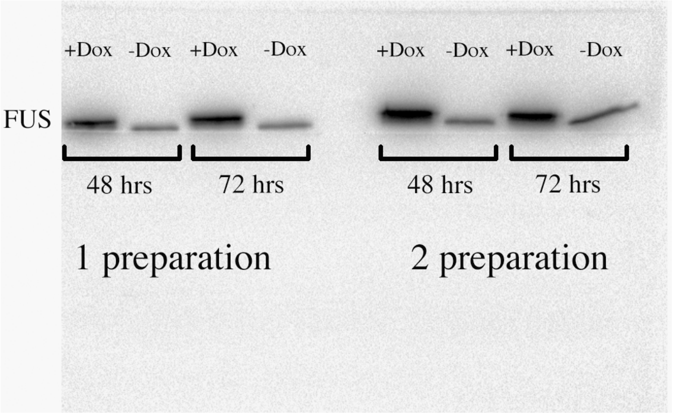

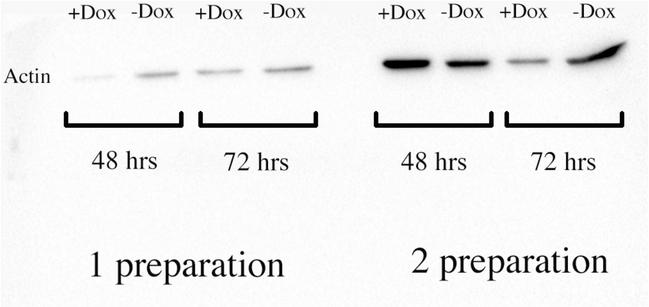

Suppl. Figure 2.
Western blot showing FUS and β-actin levels at 48 and 72 hrs upon doxycycline stimulation in mNPsc. Samples from two different preparations run on the same gel and blotted are shown. Membrane was cut for incubation with different antibodies not relevant for the present study.

Increased FUS levels in astrocytes leads to astrocyte and microglia activation and neuronal death

Maria Antonietta Ajmone Cat ,Angela Onori, Camilla Toselli, Eleonora Stronati, Mariangela Morlando, Irene Bozzoni, Emanuela Monni, Zaal Kokaia, Giuseppe Lupo, Luisa Minghetti, Stefano Biagioni, Emanuele Cacci

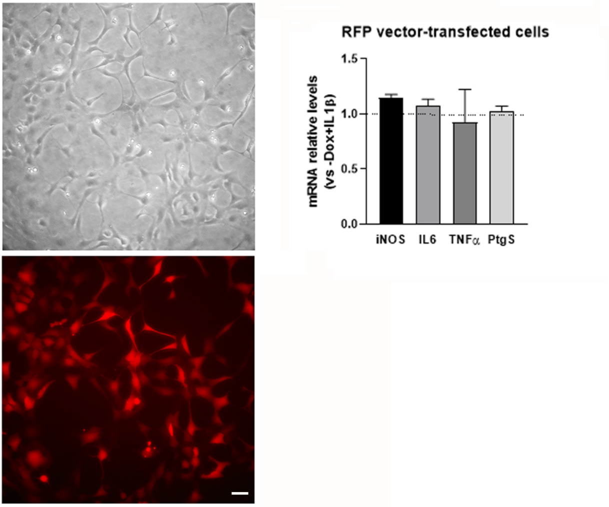

Suppl. Figure 3.
(A,B) Contrast phase and fluorescent representative microphotographic field of RFP cells upon doxycycline treatment (scale bar 20 µm).
(C) RT-PCR analysis of the expression of inflammatory genes following IL1β stimulation in RFP transfected cells induced or not with Dox to express RFP.
Data are expressed as the fold change in gene expression in Dox-induced RFP expressing cells (+Dox+IL1β) normalized to the endogenous gene (β-actin) and relative to non-induced cells (-Dox+IL1β), as assessed by using the 2-ΔΔCt method. (n = 3, in triplicate). Dox-dependent induction of RFP expression did not modify the response to IL1β compared to non-induced cells (-Dox+IL1β).

Increased FUS levels in astrocytes leads to astrocyte and microglia activation and neuronal death

Maria Antonietta Ajmone Cat ,Angela Onori, Camilla Toselli, Eleonora Stronati, Mariangela Morlando, Irene Bozzoni, Emanuela Monni, Zaal Kokaia, Giuseppe Lupo, Luisa Minghetti, Stefano Biagioni, Emanuele Cacci

Suppl Fig 4

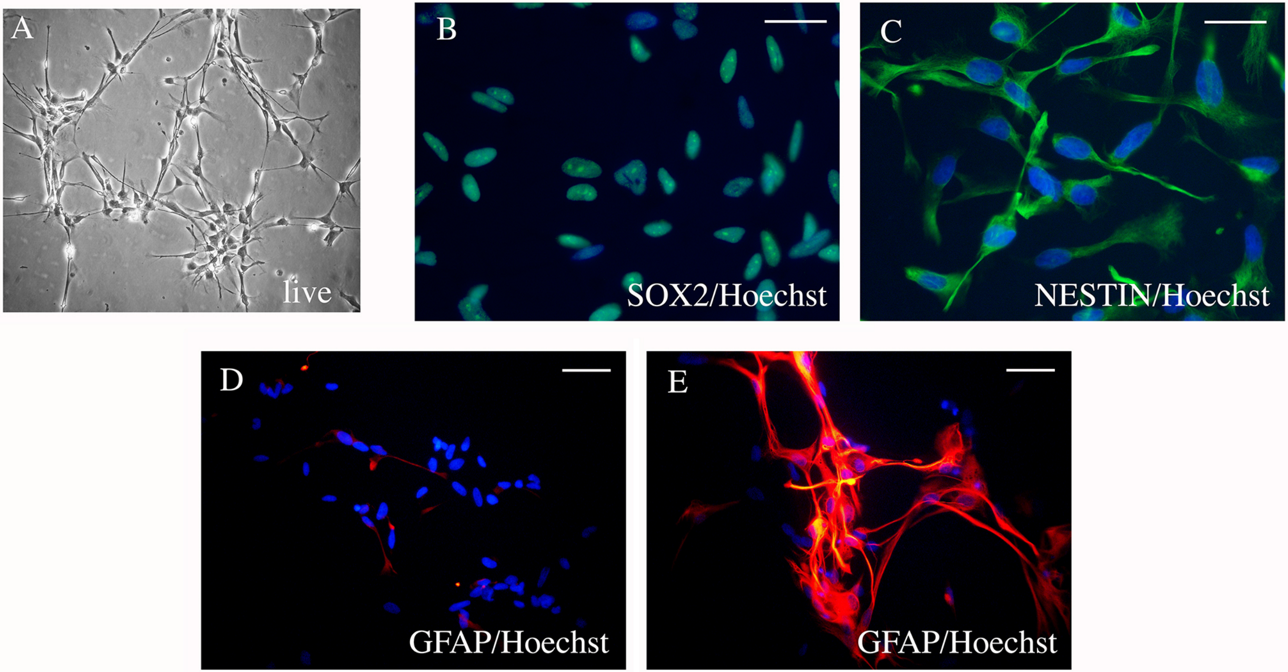

Suppl. Figure 4.
Immunofluorescence analysis of the expression of neural progenitor- and differentiation- markers in hNPsc, under different culture conditions.
(A) Representative contrast phase picture of hNPsc cells under expansion conditions.
(B-D) hNPsc, grown in expansion medium (+EGF/+bFGF), expressed the typical neural progenitor cell markers Sox2 (B) and nestin (C).
Note that under proliferative conditions GFAP was barely detectable (D). (E) Upon growth factors removal (-EGF/-bFGF) and treatment with BMP-4 for 6 days, hNPsc generated a highly enriched population of cells with typical astrocyte-like morphology, expressing GFAP. Scale bar 20 µm.

Increased FUS levels in astrocytes leads to astrocyte and microglia activation and neuronal death

Maria Antonietta Ajmone Cat ,Angela Onori, Camilla Toselli, Eleonora Stronati, Mariangela Morlando, Irene Bozzoni, Emanuela Monni, Zaal Kokaia, Giuseppe Lupo, Luisa Minghetti, Stefano Biagioni, Emanuele Cacci

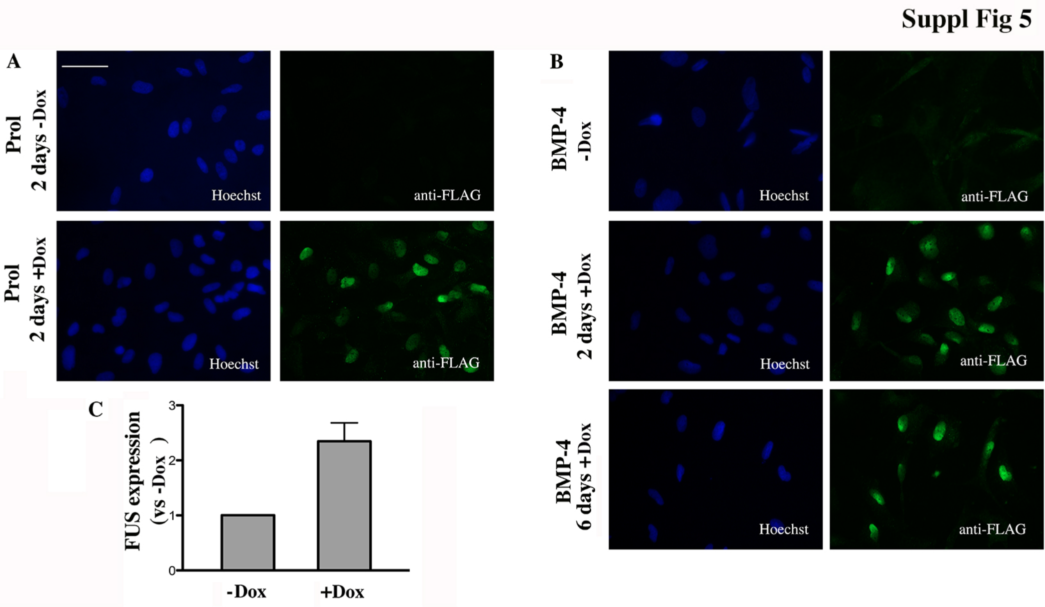

Suppl. Figure 5.
Doxycycline-dependent expression of exogenous WT-FUS in human neural progenitor cells.
(A) hNPsc cells, electroporated with a plasmid allowing for the inducible expression of WT-FUS, and selected with puromycin,showed expression of the transgene (detected by an antibody directed versus the FLAG sequence of human WT-FUS) when maintained for 48 hrs in the presence of doxycycline (+Dox). (B) Importantly, FUS expression was retained also in differentiated cells maintained for two or six days in the absence of growth factors (-EGF/-bFGF) and treated with human recombinant BMP-4. Scale bar 20 μm. (C) FUS expression was increased of about 2.5 folds in cells treated with doxycycline and differentiated for 6 days in the presence BMP-4, as assessed by RT-PCR analysis.
